# Physical Activity during Pregnancy and Risk of Gestational Diabetes Mellitus: A Meta-Review

**DOI:** 10.3390/life14060755

**Published:** 2024-06-13

**Authors:** Carmen Rute-Larrieta, Gloria Mota-Cátedra, Juan Manuel Carmona-Torres, Victoria Mazoteras-Pardo, Esperanza Barroso-Corroto, Carlos Navarrete-Tejero, Michail Zografakis-Sfakianakis, Athina Patelarou, Maria Manuela Martins, Ana da Conceinçao Alves Faria, José Alberto Laredo-Aguilera

**Affiliations:** 1 Hospital Universitario Severo Ochoa, Leganés, 28914 Madrid, Spain; c.rute@salud.madrid; 2 Hospital Universitario de Toledo, 45007 Toledo, Spain; gmota@sescam.jccm.es; 3 Grupo de Investigación Multidisciplinar en Cuidados, Universidad de Castilla-La Mancha, 45071 Toledo, Spain; esperanza.barroso@alu.uclm.es (E.B.-C.); josealberto.laredo@uclm.es (J.A.L.-A.); 4 Facultad de Fisioterapia y Enfermería, Universidad de Castilla-La Mancha, Campus Tecnológico Fábrica de Armas, Edificio 6. Despacho 1.4, Avd. Carlos III s/n, 45071 Toledo, Spain; carlos.navarrete@uclm.es; 5 Facultad de Enfermería de Ciudad Real, Universidad de Castilla-La Mancha, 13071 Ciudad Real, Spain; victoria.mazoteras@uclm.es; 6 Department of Nursing, Faculty of Health Sciences, Hellenic Mediterranean University, 71401 Crete, Greece; mzografakis@hmu.gr (M.Z.-S.); apatelarou@hmu.gr (A.P.); 7 Abel Salazar Biomedical Sciences Institute, University of Porto, 4099-002 Porto, Portugal; mmmartins@icba.up.pt (M.M.M.); up201903094@up.pt (A.d.C.A.F.)

**Keywords:** pregnancy, physical activity, gestational diabetes mellitus

## Abstract

Background: Nowadays, pregnant women require more individualized attention in their assistance process during pregnancy. One of the aspects that requires the most focus is the suitability of carrying out physical activity. The objective of this meta-review is to find out the effects of physical activity during pregnancy on the incidence of GDM compared to women who do not perform physical activity. Methods: A search was conducted in Cochrane, CSIC, Ebscohost, Proquest, Pubmed, Scielo, and Scopus. The search focused on systematic reviews and meta-analyses published in the last five years. The AMSTAR-2 scale was used as a quality assessment tool for the final sample. Results: A total of 18 systematic reviews and meta-analyses were included. Sixteen of them found out that physical activity during pregnancy has preventive effects for GDM compared with women who lacked physical activity. Among the studies, we found a reduction in the risk of GDM of between 24% and 38% and odds ratios ranging between 0.39 and 0.83 calculated for a 95% CI. Only two studies did not find statistically significant effects. Other variables such as type and duration of physical activity, overweight and obesity, gestational age, etc., were also considered. Conclusions: Physical activity prevents the incidence of GDM. The main characteristics that enhance this preventive effect are starting at the initial stages of pregnancy and maintaining during the whole pregnancy as well as combining strength and aerobic exercise at a low to moderate intensity.

## 1. Introduction

Pregnancy is considered a natural process of the female body that involves physiological, emotional, and social alterations [1,2]. Prenatal care such as medical check-ups, diagnostic tests, and consultations with the midwife is focused on enabling the woman to manage the different aspects of her pregnancy. Among these aspects, one of the ones that has evolved the most in recent years is the practice of physical activity during pregnancy [2].

Physical exercise is a form of planned, structured, and repetitive physical activity performed to improve health and/or fitness [3]. There is a great deal of scientific evidence of the benefits of physical activity at all stages of human life. According to the Physical Activity Guidelines for Americans, among the benefits of physical activity are the following: immediate benefits such as improved cognitive skills, decreased anxiety, a decreased risk of depression, and improved sleep quality [3]. In addition, the practice of physical activity reduces the risk of cardiovascular diseases, which are one of the leading causes of death worldwide [4], and reduces the risk of type 2 diabetes and metabolic syndrome and the risk of developing various types of cancer [3,5]. Despite the benefits of physical activity, one in four adults does not reach the levels of recommended physical activity by WHO [6,7]. Specifically in women, these benefits are also indisputable, having positive effects on the immune, hemostasis, metabolic, and hormonal systems in the different stages of their physiological development [8,9].

Pregnancy is another of those physiological stages in the development of women; however, for many years, pregnant women have been concerned about the possible risks associated with physical activity during pregnancy and have not been adequately counseled about physical activity during this stage [10]. They were advised not to engage in physical activity for fear of reducing their placental circulation and causing harmful consequences for the fetus. This is why, for a long time, when women have known they were pregnant, they have abandoned, reduced, or refused to carry out physical exercise [11].

In recent years, the situation has changed, improving the physical activity–pregnancy relationship and making it possible to prescribe physical activity in a way that guarantees the maximum benefits and minimum risks for the mother and fetus [12]. Taking advantage of pregnancy as a special stage to promote healthy lifestyles that last over time and promote a better state of health, different organizations recommend that the practice of physical activity during pregnancy, in the absence of medical pathologies that advise against it, should not be too far removed significantly from the recommended for the general population [4,13], recommending at least 150 min of moderate-intensity aerobic physical exercise per week in combination with muscle-strengthening activities and respecting safety considerations when doing so [4].

There are several pregnancy-related disorders for which physical activity can reduce the risk of occurrence, including hypertensive disorders during pregnancy, which affect up to 10% of pregnancies, with preeclampsia being one of the main causes of maternal morbidity and mortality [14]. Other disorders associated with the lack of physical activity in pregnancy include excessive weight gain [15], complications in childbirth, neonatal problems [10], and postpartum depression [16].

Gestational diabetes mellitus, which is defined as new-onset maternal hyperglycemia in pregnancy that resolves after birth [17], is one of the most prevalent diseases in the modern world, a consequence of factors such as obesity, inadequate nutrition, or sedentary lifestyles [18]. In addition, it increases the risk of developing diabetes after pregnancy [19], and there is a possibility of fetal macrosomia, hyperbilirubinemia and hypoglycemia, infections, and obstetric trauma, also increasing the risk of complications during pregnancy and delivery. Physical activity prevents gestational diabetes mellitus by improving glycemic control, insulin resistance, and weight control during pregnancy [20].

It has been proven that the practice of physical activity is vital at all stages of life; however, when pregnancy arrives, doubts continue to arise about the suitability of physical exercise, as well as the frequency and intensity, finding low levels of physical exercise in women during pregnancy [21]. Physical activity during pregnancy reduces the likelihood of some of these disorders, and studies have shown the positive effects of physical activity interventions on the physical and psychological health of pregnant women [22]. Although during pregnancy there are complications related to a lack of physical activity that can lead to premature births [23], macrosomic [24] or low birth weight babies [25], or poor fetal vascularization [26] and the development of other maternal problems such as the one discussed in this review, pregnant women continue to receive contradictory ideas from health professionals responsible for monitoring their pregnancy about the practice of physical activity [27]. Also, studies show that pregnant women continue to spend most of their time in sedentary behavior [28]. There are no clear recommendations on the use of physical activity to reduce the incidence of GDM, and there is a wide range of studies and reviews on the subject with different conclusions [29,30,31].

For all these reasons, a review of the most current publications is necessary to establish unanimous criteria when making physical activity recommendations to pregnant women and, more specifically, about the case of specific and evidence-based recommendations to prevent the risk of developing GDM.

## 2. Method

### 2.1. Research Question

To obtain useful results from the search, the PICO question was developed (Table 1). The aim was to find out what effect physical activity during pregnancy has on the occurrence of GDM during pregnancy compared with women who do not engage in physical activity.

### 2.2. Information Resources

The search was conducted in seven databases (MEDLINE (via PubMed), Scopus, Proquest, EBSCOHOST, SCIELO, Cochrane Library, and CSIC) to identify systematic reviews published between January 2018 and January 2024. This comprehensive search was conducted between December 2022 and February 2024.

In addition, this review has been registered in PROSPERO (York, UK) with the code CRD42023434022.

### 2.3. Search Strategy

This meta-review followed the Preferred Reporting Items of Systematic Reviews and Meta-Analyses (PRISMA) guidelines [32]. The search algorithm used the combined MeSH terms in the following way: “pregnancy” AND “physical activity”. These descriptors were chosen because of their relation to the object of study. A generic search string was used to obtain greater sensitivity and subsequently manually eliminate studies that did not meet the inclusion criteria. Table 1 shows the PICO criteria.

### 2.4. Selection Criteria

The articles obtained in the initial search were subjected to the selection criteria determined for this study. The inclusion and exclusion criteria were established as shown in Table 2.

### 2.5. Study Selection

The review and selection of studies was carried out in duplicate by two authors independently and they extracted the data from the eligible studies. The initial selection was made by reading the title and abstract of all the articles resulting from the bibliographic search in the databases. The full text of the articles was then reviewed to determine compliance with the inclusion and exclusion criteria. Any discrepancies in the cross-checks were resolved by a third reviewer and by discussion among all the participating authors.

### 2.6. Quality Assessment of Studies

The quality of the included systematic reviews was assessed using the Assessment of Multiple Systematic Reviews (AMSTAR-2 (Ottawa, ON, Canada)) [33], which uses 16 items to assess the overall confidence in the results of the review (ranging from “high” to “critically low”) and proposes a scheme for interpreting the weaknesses of the different sections (Table 3).

### 2.7. Analysis of the Obtained Data

To extract the data from each review, a table was prepared in which the following aspects were included: the first author and year of publication, databases in which the search was performed, the number of studies included in each review and their design, the total sample of the review, the intervention performed and control, the number of studies included that have results of the effects of physical activity during pregnancy on the prevention of GDM and their design, the surname of the principal investigator of each study included, and the inclusion, or not, of a meta-analysis (Table 4).

## 3. Results

### 3.1. Study Characteristics

**Figure 1:**
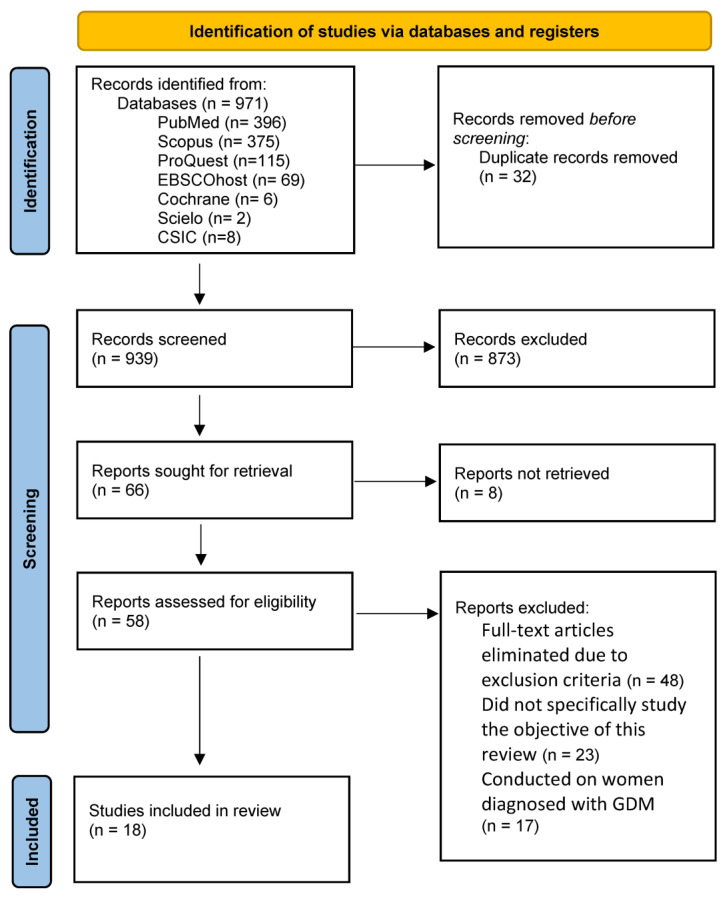
PRISMA flowchart.

The search retrieved 971 scientific articles, of which 873 were excluded by title and abstract. Next, 32 duplicate articles were eliminated. After the full-text close reading, 48 articles were eliminated for not meeting the inclusion criteria. Finally, 18 systematic reviews were included in this study (Figure 1). The characteristics of the included reviews can be seen in Table 4.

**Table 4:** Characteristics of included systematic reviews and design of included studies with effects of physical activity during pregnancy on the prevention of GDM.

First Author (Year)	Database Search Search Period	Design of Included Studies, *n*	Total Sample, *n* Intervention/Control	Design of the Included Studies with Results in the Object of Study	The First Author of the Studies Included in Each Review	Meta-Analysis
Davenport (2018) [34]	MEDLINE, EMBASE, PsycINFO, Cochrane Database of Systematic Reviews, Cochrane Central Register of Controlled Trials, EBSCO, Scopus, Web of Science Core Collection, ClinicalTrials.gov, Trip Database. Until 6 January 2017.	Randomized Control Trials (RCT) No RCT Cohort study Cross-sectional studies Case–control studies *n* = 106	273,183 pregnant women. Physical exercise (frequency, intensity, duration, volume or type). No exercise, different frequency, intensity, duration, volume or type and exercise in a different trimester.	RCT *n* = 65 Non-randomized interventions, *n* = 9 Cohort studies *n* = 13 Cross-sectional studies *n* = 11 Case–control studies, *n* = 8	Barakat, Callaway, Codero, Elden, Garnaes, Guelfi, Ko, Kon, Nobles, Okido, Oostdam, Price, Renault, Rodríguez, Ruiz, Seneviratne, Simmons, Tomic, Ussher, Wang	Yes
Ming (2018) [35]	Web of Science Scopus (Pubmed, MEDLINE, Embase) ClinicalTrials.gov Cochrane BibliotRCT From January 1994 to June 2017	RCT *n* = 8	3256 pregnant women. Exercise performance (type, frequency, duration, and intensity)/standard prenatal care.	RCT *n* = 8	Barakat, Cordero, Stafne, Tomiç, Ruiz	Yes
Davenport (2018) [36]	Medline, EMBASE, PsycINFO, Cochrane Database of Systematic Reviews, Cochrane Central Register of Controlled Trials, Scopus, Web of Science Core Collection, CINAHL Plus, Child Development and Adolescent Studies, Educational Resource Information Center, SportDiscus, ClinicalTrial.gov. By 6 January 2017.	RCT Non-randomized interventions Cohort studies Cross-sectional studies *n* = 58	8699 pregnant women without contraindications to exercise. Exercise interventions (frequency, intensity, duration, volume, type) and mixed interventions of exercise and diet/no exercise or different type, frequency, intensity, volume, or duration.	RCT *n* = 31 Non-randomized interventions *n* = 7 Cohort studies *n* = 8 Cross-sectional studies *n* = 12	Artal, Artal, Bessinger, Bonnen, Clapp, Clapp, Giroux, Halse, Jovanovic, Lotgering, McMurray, Mottola, Ruchat, Soultanakis	Yes
Muhammad (2021) [37]	Pubmed/Medline, BibliotRCT Cochrane, Web of Science, EMBASE, CINAHL. Until 31 August 2019.	RCT *n* = 11	507 pregnant women with obesity or overweight. Supervised exercise/unspecified control group.	RCT *n* = 5	Wang, Garnaes, Garnaes, Ong, Daly	Yes
Nasiri Amiri (2019) [38]	Medline, Cochrane BibliotRCT, Pubmed, Scopus, Web of Science, Embase, CINAHL. 1 January 2008 to 30 May 2018.	RCT *n* = 8	1441 overweight and/or obese pregnant women. Exercise and routine prenatal care/routine prenatal care.	RCT *n* = 8	Garnaes, Barakat, Niham Daly, Oostdam, Wang, Simmons, Seneviratn, Callaway	Yes
Cremona (2018) [39]	Medline, Pubmed, Scopus, Cinahl, Cochrane BivliotRCT, Embase, Maternal and Child Health Database. April 2018.	RCT *n* = 12	878 pregnant women with GDM or at risk for GDM. Exercise interventions/standard Care.	RCT *n* = 6	Ruchat, Ong, Callaway, Guelfi, Wang, Garnaes	No
Guo [40] (2019)	Pubmed, Scopus, Cochrane. Inception: 15 July 2017.	RCT *n* = 47	5883 pregnant women with or without GDM in exercise-only interventions. Diet and exercise interventions/standard care	RCT *n* = 19	Rakhshani, Tomiç, Renault, Barakat, Ruiz, Wang, Garnaes, Bisson, Nobles, Oostdam, Barakat, Guelfi, Kong, Stafne, Callaway, Sneviratne, Price, Ko, Barakat	Yes
Tang (2022) [41]	Medline, Embase, Pubmed, Central, CINAHL. From the start until 30 June 2022.	RCT *n* = 46	1991 pregnant women in physical activity-only intervention. Interventions to prevent GDM (physical activity, diet, probiotic intervention, or combination of these)/placebo or standard care.	RCT *n* = 14	Peléz, Barakat, Wang, Da Silva, Seneviratne, Guelfi, Shuang, Cordero, Hayes, Stafne, Oostdam, Barakat, Vinter, Harreiter	Yes
Wu (2022) [42]	Pubmed. BibliotRCT Cochrane, Web of Science and Embase. From inception to 29 March 2021.	RCT *n* = 23	8877 pregnant women who are obese or overweight. Diet, physical activity, medication, or combination of all/usual care	RCT *n* = 2	Wang, Daly	Yes
De Castro (2022) [43]	Pubmed, Scopus, Scielo. From October 2020 to January 2021.	RCT *n* = 31	7560 pregnant women. Specialist-supervised group exercise interventions/regular care, regular care with other types of group intervention, and physical activity interventions with different physical intervention programs.	RCT *n* = 7	Sagedal, Barakat, Charkamyani, Cordero, Perales, Peláez, Barakat	No
Makaruk (2019) [44]	Ebscohost, health Source, Master File Premier, MEDLINE, SportDiscus. 2–8 July 2018.	RCT EC *n* = 10	3770 healthy pregnant women with no contraindications to exercise. Exercise program (type, intensity, duration, and frequency supervised by an exercise specialist in pregnant women/unspecified control group.	RCT y EC *n* = 10	Barakat, Oosdamet, Stafne, barakat, Cordero, Barakat, Garnaes, Barakat, DaSilva, Wang	No
Bennet (2023) [45]	Cochrane Library, CINAHL, Embase, Medline. From inception to 29 September 2022.	RCT *n* = 20	6732 pregnant women without contraindication to exercise and without GDM or DM1 or DM2. Physical activity programs (type, intensity, frequency, and duration)/women receiving standard prenatal care and not participating in any structured physical activity program.	RCT *n* = 20	Barakat, Barakat, Barakat, Barakat, Barakat, Cordero, Daly, Peláez, Price, Rakhshani, Ruiz, Tomic, Wang, Bisson, Da Silva, Garnaes, Guelfi, Oostdam, Stafne, Usher	Yes
Díaz Burrueco 2021 [46]	EMBASE, Cochrane Central Register of Controlled Trials, Medline, CINAHL. From March 2019 to April 2019 and from April 2020 to May 2020.	RCT Control clinical Trials	6857 healthy pregnant women. Physical activity intervention (aerobic, strength training, yoga, cycling, walking, aquatic exercise, and supervised exercise/Healthy pregnant women with standard prenatal care.	RCT *n* = 11	Barakat, Barakat, Bisson, Cordero, Da Silva, Daly, Garnaes, Guelfi, Perales, Seneviratne, Wang	Yes
Mijatovic-Vukas (2018) [47]	Pubmed, Medline, CINAHL/EBSCO, ScienceDirect/EMBASE. October 2015 to February 2017.	Observational studies *n* = 40	30,871 pregnant women. Physical activity before and in early pregnancy/no physical activity.	Observational studies *n* = 12	Badon, Chasan-Taber, Chasan Taber, Currie, Dempsey, Dye, Morkrid, Oken	Yes
Paulsen (2023) [48]	MEDLINE (via Pubmed), EMBASE (via Ovid), Cochrane Central Register of Controlled Trials, Web of Science, and SPORTDiscus. 22–23 February 2022.	RCT *n* = 20	6448 healthy pregnant women. Physical activity during pregnancy/no physical activity, standard prenatal care.	RCT *n* = 18	Daly, Rakhshani, Barakat, DeOliveiraMelo, País, Hui, Peláez, Seneviratne, Mcdonald, Price, Sagedal, Cordero, Wang, GinardaSilva, Ruiz, Tomic	Yes
Zhang (2023) [49]	Pubmed, EMBASE, Web of Science, China. Knowledge Network, Wangfang and WeiPu.	RCT. *n* = 11.	3087 healthy pregnant women. Performance of aerobic physical exercise/non-performance of physical exercise and standard prenatal care with conventional prenatal activities.	RCT *n* = 10	Rakhshani, Wang, Chung, Chunlan, Yan, Bruno, Barakat, Ginar da Silva, Tomic, Deng, Barakat	Yes
Tsironikos (2023) [50]	PubMed, Cochrane Library Central Register of Controlled Trials (CENTRAL), and Scopus. August 2022.	RCT *n* = 41	3690 pregnant women with a high risk of GDM. Preventing GDM among high-risk women of any lifestyle intervention, including either nutrition/PA interventions/combined diet-plus-exercise interventions that are implemented during the gestational period.	RCT *n* = 31	Do Nascimento, Oostdam, Price, Barakat, Ruiz, Nobles, Bisson, Seneviratne, Perales, Krohmn Garnæs, Guelfi, Wang, Daly, Luoto, Vinter, Harrison, Petrella, Dodd, Hui, Poston, Koivusalo, Bruno, Kennelly, Chan, Ferrara, Lin, Li, Liu, Ding, Sadiya	Yes
Quotah (2024) [51]	MEDLINE, EMBASE, and the Cochrane Central Register of Controlled Trials. February 2023.	RCT *n* = 84	22,568 pregnant women or women in the preconception period. Behavioral (diet/PA/diet and PA) and/or supplements and/or pharmacological intervention/no intervention, standard or placebo.	RCT *n* = 44	Peccei, Van Horn, Ferrara, Vinter, Sartorelli, Gonzalez-Plaza, Kennelly, Poston, Bogaerts, LeBlanc, Phelan, Dodd, Petrella, Liu, Bruno, Motahari-Tabari, Gallagher, Ding, Eslami, Parat, Herring, Phelan, Roeder, Korpi-Hyövälti, Luoto, Wang, Lin, Chan, Deng, Koivusalo, Sadiya, Mohsenzadeh-learn, Harrison, Wang, Daly, Bisson, Garnæs, Kong, Seneviratne, Callaway, Guelfi, Rakhshani, Oostdam	Yes

The systematic reviews included in this meta-review cited 196 randomized controlled trials (RCTs), 16 non-randomized interventions (non-RCTs), 21 cohort studies, 23 cross-sectional studies, eight case–control studies, and 12 observational studies. In addition, 10 other studies, RCTs and cohort studies, were included, among which it was not specified which type of study they belonged to. In addition, of the total number of systematic reviews used to perform this meta-review, 15 included a meta-analysis [34,35,36,37,38,40,41,42,45,46,47,50,51,52,53,54] and 3 did not [39,43,44].

### 3.2. Type and Duration of Physical Activity

Four of these interventions had in common that they were exercises supervised by specialists [43,44,45,46]. De Castro et al. [43] studied primarily group physical activity interventions that had an average duration of 30 to 60 min and a frequency of two to four times a week. The out-of-water interventions consisted of a warm-up period with low-impact aerobic exercises such as walking and dancing followed by resistance exercises and ending with stretching; the aquatic exercises included resistance training and stretching. Makaruk et al. [44] included studies that mostly included exercise programs in which each session consisted of warm-ups lasting between 5 and 12 min, and the main part of the exercise had durations of between 20 and 60 min and consisted of both aerobic and resistance exercises and muscle toning. Aquatic exercises were also included in some of their studies. The stretching period lasted between 5 and 12 min depending on each study. Díaz-Burrueco et al. [46] analyzed some supervised studies whose duration varied between 50 and 60 min, had a regularity of three times per week, and consisted of aerobic, strengthening, and guided stretching exercises.

### 3.3. Overweight and Obesity

On the other hand, three other studies used women who were overweight and/or obese as their population [37,38,42]. In addition, the reviews carried out by Guo et al. [40] and Bennet et al. [45] also studied this. The duration, intensity, and type of exercise among the three studies conducted on women with overweight and/or obese [37,38,42] varied among the different articles that were part of the reviews.

### 3.4. Gestational Age and Physical Activity

There were six studies [35,38,40,45,46,47] that also studied interventions in certain trimesters of pregnancy. Thus, 17 studies out of the 40 that made up the systematic review of Mijatovic-Vukas et al. [47] investigated the relationship between physical activity and the risk of GDM, and 10 of them evaluated physical activity levels in early pregnancy (first trimester). Nasiri-Amiri et al. [38] also studied populations in the first or second trimester of pregnancy. Díaz Burrueco et al. [46] evaluated interventions performed in the second trimester or during the second and third trimester of pregnancy, with few studies applying the intervention only in the first trimester or third trimester and only one in all three trimesters. In the meta-analysis by Ming et al. [35], seven of the eight included clinical trials performed the intervention during all three trimesters of pregnancy. Bennet et al. [45] compared the intervention initiated during the first trimester with the intervention initiated after the first trimester. The review of Guo et al. [40] consisted of physical activity interventions initiated between weeks 7 and 20 of pregnancy (first and second trimester).

### 3.5. Frequency and Intensity of Physical Activity

Davenport et al. [34], in their systematic review, included interventions whose frequency ranged from 1 to 7 days per week, the exercise duration ranged from 10 to 90 min, and types of exercise included walking, swimming, cycling, water aerobics, resistance training, yoga, and pelvic floor muscle training. In all of the studies that were part of the review of Ming et al., 27 adopted complete light- to moderate-intensity exercise programs with a frequency of three times a week and a duration of each session between 35 and 60 min. In the review of Davenport et al. [36], glucose responses to acute and chronic exercise in pregnancy were studied for interventions ranging in frequency from 1 to 7 days per week, with a duration of 15 to 60 min per session, and the types of exercise included were aerobic exercise, yoga, resistance training, and pelvic floor muscle training. Of the thirteen studies in the review of Cremona et al. [39], two analyzed strength exercise, eight aerobic exercises, and two a combination of aerobic exercise with strength exercise (one study was finally omitted due to poor adherence to the intervention). Guo et al. [40] and Tang et al. [41] did not specify the characteristics of the physical activity interventions. Bennet et al. [45] reported different exercise volumes in eighteen of their studies.

The most relevant results of the different studies are summarized in Table 5.

## 4. Discussion

Carrying out physical activity in pregnant women seems to have a protective effect against the appearance of GDM. As observed in the results of this meta-review, the studies support the idea that in women who carried out a physical activity program compared to those who were sedentary, the incidence of GDM was significantly lower [34,35,36,38,39,40,41,43,44,45,46,47,48,49].

### 4.1. Overweight and Obese Women

Of the three reviews that considered only overweight and obese pregnant women as a population, two did not observe any significant effect of physical activity intervention on the prevention of GDM [37,42]. Nasiri-Amiri et al. [38] observed that exercise activities, in overweight or obese women, do not have a significant effect on the overall incidence of GDM, but considering the effect measure, the incidence of GDM is 24% lower in the intervention group than in the control group. An important aspect to take into account regarding this difference in results between the studies conducted by the authors of [37,42] and Nassiri-Amiri et al. [38] is that in the review conducted by Wu et al. [42], only 2 of the 23 studies studied the physical exercise-only intervention, the others being other types of lifestyle interventions, and this sample of pregnant women was considerably smaller than the one used by Nasiri- Amiri et al. [38]. Muhammad et al. [37] also used a much smaller sample size than the one used by Nasiri-Amiri et al. [38], which may explain the difference in the results. Also, Tsironikos et al. [50] find significant effects of physical activity on the prevention of GDM, although their study includes both RCTs with women at risk of GDM and overweight or obese women and does not study gestational overweight with physical activity.

Nevertheless, 24 studies from the systematic review of Guo et al. [40] also targeted overweight and/or obese women and concluded that BMI fails to predict the effectiveness of the intervention in preventing GDM, although it should be noted that this review has a critically low quality, as well as Bennet et al.’s [45], where the results of some studies also contemplated the effects of the intervention in women with a BMI ≥25, observing no significant differences between the group that performed physical activity versus the group that did not in terms of the incidence of GDM, with a critically low quality as well. In parallel, in the review of Paulsen et al. [48], meta-regression was performed on BMI, showing that the beneficial effect of physical exercise was greater in women with a lower BMI. However, it is important to point out that these three authors found, as the main results, preventive effects of physical activity on the appearance of GDM in the case of pregnant women who were not overweight or obese, as did the studies in this meta-regression [34,35,36,38,39,40,41,43,44,45,46,47].

Ultimately, four studies found no significant relationship between physical activity in overweight and obese women and the prevention of GDM [37,40,42,45], coinciding with the conclusions of other authors such as Chatzakis et al. [57] for which exercise interventions also produced no effect for the prevention of GDM in overweight pregnant women, using a larger sample size than the rest. Nasiri-Amiri et al. [38], with their results and sample size, followed the line of Pascual Morena et al. and Du et al. [31,58], who concluded that physical exercise reduced the incidence of GDM in overweight and obese women by 29%, although this author related this mainly to the weight loss effect of physical activity in overweight and obese pregnant women. Other authors such as Dipietro et al. [59] who studied in parallel the effects of physical activity during pregnancy on weight gain and the incidence of GDM found that the evidence was strong for an inverse association between physical activity and excessive gestational weight gain, as well as strong evidence for an inverse relationship between physical activity in pregnancy and the incidence of GDM. Similarly, Muktabhant et al. [30] state that there is high-quality evidence indicating that exercise during pregnancy may reduce the risk of excessive weight gain during pregnancy, and other authors such as Sun et al. [60] confirm the unequivocal association between excessive gestational weight gain and an increased risk of developing GDM, so we can affirm that weight loss through physical activity reduces the possibility of developing GDM as confirmed by the authors of [31,38,58].

In addition, factors that preclude physical activity during pregnancy must be considered, as the study by La Verde M. et al. [61] found an increased incidence of GDM after SAR-CoV-2 confinement and excessive weight gain during pregnancy related to a lack of physical activity.

### 4.2. Specialist-Supervised Interventions

Among the findings of this review, Castro et al. [43] found that the proportion of women diagnosed with GDM was higher in the control group, as well as lower figures in the oral glucose tolerance test in the intervention group. Similarly, Díaz-Burrueco et al. [46] concluded that supervised physical activity interventions have a protective effect on GDM compared with the control group, as well as Makaruk et al. [44] and Paulsen et al. [48]. All these systematic reviews used a large sample size and have in common that the duration of the interventions was long lasting (the minimum between 8 and 10 weeks of intervention). In addition, the sessions were carried out with similar frequencies (2–3 times a week). The results found in these studies coincide with those concluded in other publications carried out previously with similar supervised interventions [29,62,63], in which supervised physical exercise was also found to have protective effects on the occurrence of GDM and beneficial effects on postprandial glucose levels [63].

### 4.3. Time of Intervention

Analyzing the results of the studies finally included in this meta-review, Nasiri-Amiri et al. [38] found a statistically significant relationship between physical activity during the first or second trimester of pregnancy. Likewise, Bennet et al. [45] concluded that physical activity interventions performed in the first trimester of pregnancy resulted in a significant decrease in the risk of GDM, but interventions that started later did not. Guo et al. [40], whose systematic review was composed of physical activity interventions initiated between the first and second trimester of pregnancy (weeks 7 to 20), also determined that preventive physical activity interventions should begin as early as possible because the earlier they begin, the greater the preventive effect, which is consistent with the findings of Nasiri-Amiri et al. [38] and Mijatovic-Vukas et al. [47], whose research also focused on the first trimester, and the intervention also produced a reduction of between 11% and 52% in the risk of GDM. Along the same lines, Díaz Burrueco et al. [46] also found preventive effects of the intervention in the periods studied by them (second and third trimester), and Ming et al. [35] concluded that physical activity in pregnancy could reduce the risk of GDM in whichever trimester it is performed, although they report high heterogeneity in their studies. These studies show similar results to others published along the same line, such as Chen et al.’s [64], which states that fasting plasma glucose levels improve with the introduction of a physical activity intervention in the first and second trimester, and Sanabria et al.’s [65] which, as well as Ming et al.’s study [35], observed a protective effect of physical exercise in the prevention of GDM in all trimesters of pregnancy. Therefore, a protective effect is found in all three trimesters, with this effect being greater the earlier physical exercise is started.

### 4.4. Physical Activity and GDM Prevention

Physical activity performed during pregnancy has beneficial effects in preventing or significantly reducing the occurrence of GDM. The meta-analyses that described physical activity intervention in terms of the duration or frequency of intervention, such as Ming et al. [35] and Bennet et al.’s studies [45], offered more specific results in the same terms, but in general, the results found were similar in all the reviews in favor of the protective effect of physical activity during pregnancy against the incidence of GDM. Thus, Bennet et al. [45] confirmed a decreased risk of GDM in interventions that had an estimated weekly training volume >600 MET-min-week, while below these volumes, no significant differences were observed. Ming et al. [35] saw the same effects with interventions they defined as light to moderate intensity performed three times per week. Also, Davenport et al. [34] determined that mild to moderate or moderate-intensity exercises reduced the incidence of GDM, with a “High” quality of vision. This same author in a different study [36] that had a “High” level of evidence quality, which in this case focused on the variation in plasma glucose after exercise, related high volumes of physical exercise (intensity by duration) with a decrease in plasma glucose levels after exercise and showed a dose–response relationship (the greater the exercise volume, the greater the low glucose response). Along the same lines of this study, Cremona et al. [39] observed a positive impact of exercise when it was performed at least three times a week, with a duration of 40 to 45 min, coinciding with the previous results. Other authors of the present review [40,41,49] also found preventive effects of physical activity during pregnancy against GDM, without specifying exactly the characteristics of their interventions. Paulsen et al. [48], in this sense, argued that the longer the intervention lasted, the greater the beneficial effect. In short, these results coincide with those of other authors who have evaluated physical activity interventions and found a protective effect on GDM. Russo et al.’s study [66] indicated a 28% lower risk of developing GDM, Tobias et al.’s [54] also indicated that high levels of physical activity resulted in a significantly lower risk of GDM, and Zakaira et al.’s [53] concluded that physical activity protects against GDM (risk reduction between 23 and 59%) with a strong inverse relationship.

It is also important to note that some of the reviews studied compared physical activity with other interventions such as diet or pharmacological supplementation to prevent GDM without obtaining significant results compared to physical activity, which did show results [50,51].

### 4.5. Limitations and Strengths

As limitations of this meta-review, some reviews studied other types of interventions in addition to physical activity, and although only those that studied physical activity were taken into account in the results, it is necessary to consider the possibility that pregnant women, knowing their condition, can spontaneously carry out other types of interventions, for example, of the dietary type, which could also have influenced the reduction in glucose levels and therefore the prevention of this pathology. In addition, some studies did not explain exactly what characteristics of frequency, duration, and intensity the exercise interventions had to produce the result of a lower probability of the incidence of GDM, so there is little unification of the minimum characteristics that physical activity should have to produce the expected effect of the prevention of GDM when it comes to having a unanimous criterion for making specific recommendations to pregnant women. Finally, we found that only two systematic reviews of the sixteen included reached the quality score “High” according to the AMSTAR-2 tool used, the rest obtaining low scores. The AMSTAR-2 tool consists of 16 domains, some of which may be difficult to apply in practice, making the tool complex to use. In addition, some important values may be difficult to include in the assessment, which may limit the tool’s ability to fully assess the quality of systematic reviews.

As strengths of this meta-review, we can highlight the fact that only systematic reviews and meta-analyses carried out very recently (in the last five years) were included, a large number of RCTs were included in the systematic reviews under study, and the size of the samples used were large. We should also point out that two of the meta-analyses included [34,36] obtained a “High” quality evaluation with the tool AMSTAR-2 [33]. Another strength of the meta-review was that a comprehensive search of multiple databases was performed, which increased the likelihood of finding relevant studies, and the PRISMA recommendation guidelines were followed.

An additional strength of this study is that after reviewing 18 systematic reviews on pregnancy and physical activity, clear recommendations on physical activity during pregnancy can be established, based on the best scientific evidence.

### 4.6. Implications for Future Research

As a recommendation for future research, it is suggested that studies should be carried out where the implementation of the most critical points is specified to be considered of high quality and to be able to offer more reliable results. It is also recommended, for future studies, to specify in more detail the specific characteristics of the intervention in terms of the duration of the intervention, frequency of the sessions, duration of each session, and intensity of the sessions to unify the characteristics that this physical activity intervention should have when making a recommendation to a pregnant woman. On the other hand, it can also be stated that there are certain cases in which the relationship between physical activity and the prevention of GDM is not so clearly observed and there are differences between studies, as in the case of some that studied the overweight and obese population.

In addition, quality studies and reviews on the effects of physical activity in pregnant women with obesity are also needed, since there are studies that differ in the benefits of physical activity on GDM.

## 5. Conclusions

Physical activity in pregnant women prevents the onset and/or reduces the progression of GDM and improves blood glucose levels. In general, it seems that longer durations of the intervention, as well as higher levels of intensity and frequency of the intervention, always conducted with supervision and in a personalized manner, are related to better prevention results.

Based on the results, we can conclude that moderate-intensity exercise two to three times a week, lasting between 30 and 60 min and exceeding 600 MET-min/week, has protective effects against GDM.

It is important to highlight that starting physical activity early in pregnancy increases the preventive power against GDM. This prevention of GDM with physical activity is achieved with light to moderate intensity levels, combining aerobic and muscular resistance exercises.

It is highly recommended to implement physical exercise programs in primary care since the follow-up of pregnant women by the midwife, as a nursing professional in close contact with the woman throughout her pregnancy, constitutes an unequaled opportunity to infuse in women the acquisition of the practice of physical activity as a tool to promote their health during this stage of reproductive health and, at the same time, to take advantage of the opportunity to establish healthy lifestyle habits that will last in later stages of the woman’s adult life.

## Figures and Tables

**Table 1:** PICO criteria.

Population	Healthy pregnant women
Intervention	Physical activity
Comparison	Pregnant women who are not physically active
Outcome	Effects of physical activity on the prevention of GDM

**Table 2:** Selection criteria.

Inclusion Criteria	Exclusion Criteria
Systematic review and/or meta-analysis	Works that are not systematic reviews or meta-analyses
Revisions published between January 2018 and February 2023	Revisions published before 2018
Reviews aiming to study the effects of physical activity during pregnancy and the prevention of GDM	Reviews that do not specifically address the objective of this meta-review
Pregnant women	Pregnant women diagnosed with GDM
Studies published in English, Spanish, French, or Portuguese.	Studies published in other languages
Women of any age and any race or ethnicity	

**Table 3:** Ratings of systematic reviews and meta-analyses with Assessment of Multiple Systematic Reviews 2 (AMSTAR-2).

Review	1	2	3	4	5	6	7	8	9	10	11	12	13	14	15	16	Quality
Davenport (2018) [34]	Yes	Yes	Yes	Yes	Yes	No	No	Yes	Yes Partial	No	Yes	Yes	Yes	Yes	Yes	Yes	Low
Ming (2018) [35]	No	Partial Yes	Yes	Yes	Yes	Yes	No	Yes	Yes Partial	No	Yes	Yes	No	Yes	Yes	Yes	Critically Low
Davenport (2018) [36]	Yes	Yes	Yes	Yes	Yes	Yes	Yes	Yes	Yes	No	Yes	Yes	Yes	Yes	Yes	Yes	High
Muhammad (2021) [37]	Yes	Partial Yes	Yes	Yes	Yes	Yes	No	Yes	Yes	No	Yes	Yes	No	Yes	Yes	Yes	Low
Nasiri-Amiri (2019) [38]	Yes	No	Yes	Yes	Yes	Yes	No	Yes	Yes	No	Yes	Yes	Yes	Yes	Yes	Yes	Low
Cremona (2018) [39]	Yes	Partial Yes	Yes	Yes	Yes	Yes	No	Yes	Yes	No	No Meta	No Meta	Yes	Yes	No Meta	Yes	Low
Guo (2019) [40]	No	Partial Yes	Yes	No	No	Yes	No	No	Yes	No	Yes	Yes	No	Yes	Yes	Yes	Critically Low
Tang (2022) [41]	Yes	No	Yes	Yes Partial	Yes	Yes	No	Yes	Yes	No	Yes	Yes	Yes	Yes	Yes	No	Critically Low
Wu (2022) [42]	Yes	Partial Yes	Yes	Yes	Yes	Yes	No	Yes	Yes	Yes	Yes	No	Yes	Yes	Yes	Yes	Low
De Castro (2022) [43]	Yes	No	Yes	Yes Partial	No	No	No	Partial Yes	Yes	No	No Meta	No Meta	Yes	Yes	No	Yes	Critically Low
Makaruk (2019) [44]	Yes	Partial Yes	Yes	Yes	Yes	Yes	No	Yes	No	No	No Meta	No Meta	No	Yes	No Meta	Yes	Critically Low
Bennet (2023) [45]	Yes	No	Yes	Yes	Yes	Yes	No	Partial Yes	Yes	No	Yes	Yes	Yes	Yes	Yes	Yes	Critically Low
Díaz Burrueco (2021) [46]	Yes	Yes	Yes	Yes Partial	Yes	Yes	No	Yes	Yes	No	Yes	Yes	Yes	Yes	Yes	Yes	Low
Mijatovic-Vukas (2018) [47]	Yes	Partial Yes	No	Yes	Yes	Yes	No	Partial Yes	No	No	Yes	No	No	Yes	Yes	Yes	Critically Low
Paulsen (2023) [48]	Yes	Partial Yes	Yes	Yes	Yes	Yes	Yes	Yes	Yes	No	Yes	Yes	Yes	Yes	Yes	Yes	High
Zhang (2023) [49]	Yes	No	Yes	Partial Yes	No	No	No	Partial Yes	Yes	No	Yes	No	Yes	Yes	Yes	Yes	Critically Low
Tsironikos (2023) [50]	Yes	Partial Yes	Yes	Yes	Yes	Yes	No	Yes	Yes	No	Yes	Yes	Yes	Yes	Yes	Yes	Low
Quotah (2024) [51]	Yes	Partial Yes	Yes	Yes	Yes	Yes	No	Yes	Yes	No	Yes	Yes	Yes	Yes	Yes	Yes	Low

1. Did the review’s research questions and inclusion criteria include the PICO components? 2. Does the review report contain an explicit statement that the review methods were established before the review and justify any significant deviations from the protocol? 3. Did the review authors explain their decision about the study designs to be included in the review? 4. Did the review authors use an exhaustive literature search strategy? 5. Did the authors of the review perform a separate study extraction? 6. Did the authors of the review perform duplicate data extraction? 7. Did the review authors provide a list of excluded studies and justify the exclusions? 8. Did the review authors describe the included studies in sufficient detail? 9. Did the review authors use a satisfactory technique for assessing the risk of bias in the individual studies included in the review? 10. Did the review authors report the sources of funding for the studies included in the review? 11. If a meta-analysis was performed, did the review authors use appropriate methods for the statistical pooling of results? 12. If a meta-analysis was performed, did the review authors assess the potential impact of risk of bias in individual studies on the results of the meta-analysis or other evidence synthesis? 13. Did the review authors consider the risk of bias of individual studies when interpreting/discussing the results of the review? 14. Did the review authors provide a satisfactory explanation and discuss any heterogeneity observed in the review results? 15. If a quantitative synthesis was performed, did the review authors conduct an adequate investigation of publication bias (small study bias) and discuss its likely impact on the review results? 16. Did the review authors report any potential sources of conflicts of interest, including any funding received to conduct the review?

**Table 5:** Results were observed in systematic reviews between physical activity interventions in pregnant women and control.

Author/Year	Results	*p* or 95% CI	Duration/Intensity of Physical Activity	Conclusions
Davenport (2018) [34]	In 45 RCTs, prenatal exercise is associated with a 24% reduction in the chance of developing Gestational Diabetes Mellitus (GDM) compared to no exercise. Exercise-only interventions (26 RCTs) reduced the odds of developing GDM by 38% compared to no exercise. Exercise + co-interventions found no statistically significant differences. Findings in non-randomized interventions, cohort, and cross-sectional studies were consistent with findings from RCTs. Case–control studies showed no significant relationship between prenatal exercise and GDM.	95% CI 0.65–0.88 OR 0.76 I^2^ = 31% 95% CI 0.52–0.75 OR 0.62 I^2^ = 0%	600 MET-min/week of moderate-intensity exercise.	Exercise-only interventions were effective in decreasing the chances of developing GDM.
Ming (2018) [35]	Physical exercise during pregnancy significantly reduces the occurrence of GDM in normal weight women.	RR = 0.58 95% CI (0.37, 0.90), *p* = 0.01 and RR = 0.60, 95% CI (0.36, 0.98), *p* = 0.04	Light to moderate exercise for 30–60 min, three times a week, during pregnancy.	In total, 30–60 min of light–moderate-intensity exercise three times a week during pregnancy could significantly reduce the occurrence of GDM.
Davenport (2018) [36]	Six studies showed a 0.94 mmol/L reduction between before and during exercise. Dose–response relationship in which the greater the exercise volume (intensity x duration of exercise, expressed in metabolic equivalents (METs) min per session), the greater the reduction in glucose during exercise.	95% CI (−1.18, −0.70), I^2^ = 41% *p* < 0.01	The exercise ranged from 1 to 7 days per week, 15 to 60 min per session, and the types of exercise included were aerobic exercise, yoga, resistance training, and pelvic floor muscle training.	A reduction in plasma blood glucose levels was demonstrated in pregnant women without diabetes during pregnancy during, after acute prenatal exercise, and also after chronic exercise interventions.
Muhammad (2021) [37]	There was no significant association between supervised exercise performance and the incidence of GDM. Moderate heterogeneity.	RR = 0.78 95% CI (0.51, 1.19) *p* = 0.25 I^2^ = 49% *p* = 0.08	Supervised moderate 10–40 min exercise like cycling, treadmill walk/jog, strength exercise, and stretching.	No effect was observed concerning the incident MGD.
Nasiri-Amiri (2019) [38]	Moreover, 143 women in the intervention group developed GDM. There was a statistically significant relationship in those studies in which the intervention was performed in the first trimester of pregnancy, not being statistically significant in those in which it was performed in the second trimester of pregnancy.In those studies in which the intervention was performed three times a week or less, a reduction in the incidence of diabetes was observed. In studies with interventions more than three times a week, no reduction in the incidence of GDM was observed.	RR = 0.76 95% CI (0.56, 1.03) I^2^ = 50% 1° trimester 0.85, 95% CI (0.55, 1.29), I^2^ = 66%, *p* = 0.03 2° trimester 0.64, 95% CI (0.40,1.04), I^2^ = 23%, *p* = 0.27 0.59, 95% CI (0.46, 0.76) I^2^ = 0% *p* = 0.47 1.03, 95% CI (0.78, 1.35) I^2^ = 0% *p* = 0.46	Low to moderate physical activity three times a week, including aerobic, resistance, strength, and pelvic floor exercises.	Exercise activities alone in overweight or obese women had no significant effect on the overall incidence of GDM, but considering the effect measure, the incidence of GDM was 24% lower in the intervention group than in the control group.
Cremona (2018) [39]	Aerobic exercise: one study reported that the incidence of GDM was improved. Combined aerobic and strength exercise: the incidence of GDM was lower in the intervention group than in the control group.	6.1% in the study group vs. 27.3% in the control group, *p* = 0.04	Three times per week for 40–60 min at 65–75% age—predicted heart rate maximum using cycling, walking, or circuit training.	Pregnant women with obesity can improve glycemic control during pregnancy and the incidence of GDM through exercise.
Guo (2019) [40]	Exercise intervention produced a reduction in the incidence of GDM.	RR = 0.70 95% CI (0.59, 0.84)	Moderate intensity exercise for 50–60 min twice a week.	Diet and physical exercise during pregnancy were preventive measures for GDM.
Tang (2022) [41]	The physical activity intervention significantly reduced the incidence of GDM compared to the control group.	OR = 0.64 95% CI (0.46–0.88)	Physical activity in general.	Physical activity is more effective than a placebo in reducing the incidence of GDM and can be considered as an adjunctive therapy to prevent it.
Wu (2022) [42]	The intervention with physical activity yielded insignificant results, although it was the best of all the interventions studied.	RR = 0.75 95% CI (0.50, 1.11)	Personalized recommendations, supervised aerobic exercise sessions, or additional materials for physical activity advice.	Neither physical exercise nor the rest of the interventions were able to offer any significant benefit in the prevention of GDM.
De Castro (2022) [43]	The ratio of women diagnosed with GDM was higher in the control group. Physical exercise programs report a lower risk of GDM.	A higher proportion of women diagnosed with GDM in the control group. Lower figures in the oral glucose tolerance test in the intervention group.	Supervised in groups exercise of one to four times per week and an average duration of 60 min.	Significant effects of group exercise programs on various aspects of maternal health, including improved glucose tolerance.
Makaruk (2019) [44]	Two studies (Cordero et al. [55], Wang et al. [56] of physical activity interventions during pregnancy were successful in preventing GDM. The other 8 studies did not prove effective in reducing GDM.	Wang [56]: *p* < 0.01 Cordero [55]: *p* = 0.009	Moderate physical activity (duration 40–60 min), and each session was divided into the warm-up, main part, and cool-down.	This article reveals a small number of studies that are effective for the prevention of GDM.
Bennet (2023) [45]	There was a significant 38% reduction in the risk of GDM in the group in which physical activity intervention was performed. This reduction was more significant in interventions that began within the first trimester. In those who started after the first trimester, there was no significant reduction in the risk of GDM. Significant reduction in the risk of GDM in those interventions with an estimated weekly training volume of >600 MET·min·wk^−1^ There was also a significant 49% reduction in the physical activity intervention with women who had a mean BMI of 25 kg/m^2^ at baseline.	RR 0.62, 95% CI (0.46, 0.82), *p* = 0.002, I^2^ = 62% RR = 0.57, 95% CI (0.41, 0.79), *p* = 0.001, I^2^ = 50% RR = 0.96, 95% CI (0.75, 1.22), *p* = 0.73, I^2^ = 0% RR = 0.77, 95% CI (0.60, 0.98), *p* = 0.03, I^2^ = 13% RR = 0.51, 95% CI (0.34, 0.75), *p* = 0.001, I^2^ = 33%	>600 MET/min/week.	Women who engaged in supervised physical exercise beginning in the first trimester of pregnancy were less likely to develop GDM compared to non-active women in the control group.
Díaz Burrueco (2021) [46]	The physical activity intervention, compared to the control group, has a protective effect against GDM	OR = 0.65 95% CI (0.43, 0.98) I^2^ = 48%	Supervised exercise, three times per week, duration of 60 min.	Physical activity interventions during pregnancy play a preventive role in the incidence of GDM.
Mijatovic-Vukas (2018) [47]	Physical activity in the initial stages of pregnancy is related to a reduction in the risk of GDM between 11 and 52%	RR = 0.31 95% CI = 0.12–0.79	≥150 min/week or ≥210 min/week and ≥15 or ≥21 MET hours/week.	Physical activity is a promising intervention in the prevention of GDM.
Paulsen (2023) [48]	A favorable effect is observed of performing physical exercise on not performing.Greater beneficial effect on: -older women-women with lower BMI -women who have received longer interventions -women whose intervention has been supervised by a specialist	RR = 0.66 95% CI = 0.50–0.86 I^2^ = 47.68% OR = 0.83 (0.74–0.93) *p* = 0.001, I^2^ = 0% OR = 1.10 (1.00–1.21) *p* = 0.04, I^2^ = 41.2% OR = 0.93 (0.89–0.97) *p* = 0.001, I^2^ = 3.5% OR = 0.98 (0.97–0.99) *p* = 0.002, I^2^ = 5.2%	Moderate supervised exercise three times per week, aerobic, resistance, and mind-body training.	Aerobic exercise and a combination of aerobic and mind–body exercises during pregnancy are beneficial to prevent GDM. Likewise, moderate and less intense interventions supervised by a specialist seem equally effective.
Zhang (2023) [49]	The incidence of GDM was lower in the intervention group compared to the control group and this intervention reduced the incidence rate with a significant difference between groups.	OR = 0.39 95% CI = 0.30–0.50 I^2^ = 0% *p* < 0.00001	Aerobic exercises.	Physical activity interventions during pregnancy are beneficial and effective in reducing the incidence of GDM.
Tsironikos (2023) [50]	Overall, a significant reduction in the risk of developing GDM was observed in high-risk women who received exercise interventions alone and in those who received combined diet and exercise interventions (relative risk meta-analysis). Diet interventions alone showed a risk reduction, but the result was not statistically significant.	OR = 0.64 95% CI = 0.51–0.80 I^2^ = 0% *p* < 0.0001	Exercises provided by healthcare professionals.	Physical activity during pregnancy helps reduce the risk of GDM significantly more than other interventions such as dietary changes.
Quotah (2024) [51]	Gestational diabetes was reduced in diet and physical activity, inositol, and vitamin D supplements. Subgroup analysis showed that diet and physical activity interventions were beneficial in women with ≥2 GDM risk factors while inositol supplementation was effective in women with overweight or obesity. The effectiveness of all other interventions was not statistically significant.	RR = −0.03 95% CI 0.06, −0.01 I^2^ = 58.69% RR = −0.16, 95% CI 0.25, −0.07 I^2^ = 11.23% RR = −0.17 95% CI 0.22, −0.11 I^2^ = 0.01%	Physical activity in general, combined with diet.	Diet and physical exercise combined with supplementation and pharmacological treatment help prevent GDM.

## Data Availability

The data used in this systematic meta-review are accessible through the scientific databases used.
